# Análise da Razão Neutrófilo-Linfócito como Marcador de Aterosclerose da Aorta Abdominal

**DOI:** 10.36660/abc.20220145

**Published:** 2022-11-01

**Authors:** João Victor Domiciano Martins, Rodrigo Mendes, Johnnatas Mikael Lopes, Pedro Pereira Tenório

**Affiliations:** 1 Universidade Federal de São Paulo São Paulo SP Brasil Universidade Federal de São Paulo, São Paulo, SP – Brasil; 2 Universidade Federal do Vale do São Francisco Paulo Afonso BA Brasil Universidade Federal do Vale do São Francisco – Colegiado de Medicina, Paulo Afonso, BA – Brasil

**Keywords:** Aterosclerose, Biomarcadores, Linfócitos, Neutrófilos, Fatores de Risco, Aorta

**Caro Editor,**

Lemos com grande interesse o artigo: *Razão Neutrófilo-Linfócito e Aterosclerose da Aorta Abdominal entre Indivíduos Assintomáticos* que avaliou se a razão neutrófilo-linfócito (RNL) poderia estar associada a aterosclerose da aorta abdominal (AtAA). Foi demonstrada uma associação positiva entre o aumento da RNL e AtAA ao comparar portadores com não portadores da doença. Entretanto, ao se ajustar a análise estatística para idade e fatores de risco, essa associação não permaneceu verdadeira.^1^

Está melhor estabelecido na literatura que a RNL está associada a fase aguda da aterosclerose coronariana. Além disso, é sabido que o aumento do número de neutrófilos presentes na parede da aorta é significativamente maior nas placas instáveis, rotas e/ou recentes quando comparado aos ateromas estáveis fibróticos. Estes últimos apresentam redução não somente do número de neutrófilos, como também de células dendríticas e natural killer (NK).^2^

A adoção da ultrassonografia convencional apresenta sensibilidade de 62% para detecção de oclusões arteriais com volume inferior a 8mm^4^ e apenas 25% de sensibilidade para placas carotídeas instáveis.^3,4^ Tal técnica pode ter influenciado o resultado do estudo, haja vista que placas recentes em crescimento e placas instáveis não calcificadas podem não ter sido identificadas. Uma possível solução teria sido adotar outro método de triagem. A elastografia em tempo real apresenta uma sensibilidade de 50% para a detecção das mesmas placas instáveis, ou a associação de ambos os métodos – o que teria aumentado a sensibilidade para 62,5%.^4^ A adoção de um método mais sensível de triagem poderia não apenas identificar um número maior de pacientes com processo aterosclerótico em curso, como também detectaria ateromas recentes e/ou instáveis em maior número, o que alteraria de forma significativa a estatística final.

A aterosclerose pode acometer diferentes territórios vasculares com prevalências distintas. Mesmo os fatores confundidores bem destacados, deve-se notar a alta prevalência dessa doença em outras artérias, além da aorta, em indivíduos mais velhos. Pode-se questionar a possibilidade dos pacientes de idade avançada do grupo controle, aos quais a estatística foi ajustada para idade, apresentarem processo aterosclerótico para além da aorta abdominal. Este fator de confusão poderia ser evitado com a investigação da presença de aterosclerose em outras artérias, entrando como critério de exclusão, ou que a RNL fosse abordada como um possível preditor de aterosclerose sistêmica e não apenas da aorta abdominal.

Por fim, a proporção de sexo masculino nos quintis de RNL na tabela 1 é epidemiologicamente semelhante assim como entre aqueles com/sem aterosclerose na tabela 2. Como a amostra não foi selecionada aleatoriamente, isso pode retratar apenas um maior acesso ao exame preventivo pelo sexo masculino. O que ocorre também com a variável tabagismo atual. Soma-se a isso estatísticas analíticas ausentes da modelagem multivariada como indicadores de qualidade dos modelos ajustados que impedem uma interpretação crítica do leitor quanto a validade associativa informada. Estimativa de risco dos ajustes também melhoraria a interpretação dos modelos. Além disso, em estudos com dados de desfecho (aterosclerose) e independentes (RNL) coletados ao mesmo tempo (desenho transversal), o uso de regressões logísticas superdimensiona os estimadores intervalares como o IC95%, podendo ter ocorrido nos modelos 1 e 2. A melhor indicação nestas análises são as regressões de Poisson ou Cox adaptada.^5^
