# Supplementary material for: Análise da Razão Neutrófilo-Linfócito como Marcador de Aterosclerose da Aorta Abdominal
Source: Arq Bras Cardiol. 2022 Nov 1;119(5):813–4. [Article in Portuguese] doi: 10.36660/abc.20220145 (PMC9750215; doi:10.36660/abc.20220145)
Supplement: Translation

# Translation: Analysis of the Neutrophil-Lymphocyte Ratio as a Marker of Atherosclerosis of the Abdominal Aorta

**Authors:** João Victor Domiciano Martins, Rodrigo Mendes, Johnnatas Mikael Lopes, Pedro Pereira Tenório

**Affiliations:** 1 São Paulo SP Brazil Universidade Federal de São Paulo, São Paulo, SP – Brazil; 2 Paulo Afonso BA Brazil Universidade Federal do Vale do São Francisco – Colegiado de Medicina, Paulo Afonso, BA – Brazil

**Keywords:** Atherosclerosis, Biomarkers, Lymphocytes, Neutrophils, Risk Factors, Aorta

**Dear Editor,**

We read with great interest the article: Neutrophil-Lymphocyte Ratio and Abdominal Aortic Atherosclerosis among Asymptomatic Individuals, which evaluated whether the neutrophil-lymphocyte ratio (NRL) could be associated with abdominal aortic atherosclerosis (AtAA). A positive association between the increase in NLR and AtAA was demonstrated when comparing carriers and non-carriers of the disease. However, when adjusting the statistical analysis for age and risk factors, this association did not remain true.^1^

It is better established in the literature that NLR is associated with the acute phase of coronary atherosclerosis. Furthermore, it is known that the increase in the number of neutrophils present in the aortic wall is significantly greater in unstable, ruptured and/or recent plaques when compared to stable fibrotic atheromas. The latter show a reduction not only in the number of neutrophils but also in dendritic and natural killer (NK) cells.^2^

The adoption of conventional ultrasonography has a sensitivity of 62% for detecting arterial occlusions with a volume of less than 8mm^4^ and only 25% sensitivity for unstable carotid plaques.^3,4^ Such a technique may have influenced the study's results, given that newly growing plaques and unstable, non-calcified plaques may not have been identified. A possible solution would have been to adopt another screening method. Real-time elastography has a sensitivity of 50% for the detection of the same unstable plaques or the combination of both methods - which would have increased the sensitivity to 62.5%.^4^ Adopting a more sensitive screening method could not only identify a greater number of patients with an ongoing atherosclerotic process but also detect more recent and/or unstable atheromas, which would significantly alter the final statistics.

Atherosclerosis can affect different vascular territories with different prevalence. Even though confounding factors are well highlighted, the high prevalence of this disease in other arteries, in addition to the aorta, in older individuals should be noted. One may question the possibility that elderly patients in the control group, whose statistics were adjusted for age, have an atherosclerotic process beyond the abdominal aorta. This confounding factor could be avoided by investigating the presence of atherosclerosis in other arteries, entering as an exclusion criterion, or approaching the LNR as a possible predictor of systemic atherosclerosis and not only of the abdominal aorta.

Finally, the proportion of males in the NLR quintiles in Table 1 is epidemiologically similar to those with/without atherosclerosis in Table 2. As the sample was not randomly selected, this may only portray greater access to preventive examination by males. This also occurs with the current smoking variable. Added to this are analytical statistics absent from multivariate modeling as indicators of the quality of the fitted models that prevent a critical interpretation by the reader regarding the reported associative validity. Risk estimation of the adjustments would also improve the interpretation of the models. In addition, in studies with the outcome (atherosclerosis) and independent (NLR) data collected at the same time (cross-sectional design), the use of logistic regressions overestimates interval estimators such as the 95%CI, which may have occurred in models 1 and 2. The best indication in these analyses is the adapted Poisson or Cox regressions.^5^
