# Supplementary material for: Análise da Razão Neutrófilo-Linfócito como Marcador de Aterosclerose da Aorta Abdominal
Source: Arq Bras Cardiol. 2022 Nov 1;119(5):813–4. [Article in Portuguese] doi: 10.36660/abc.20220145 (PMC9750215; doi:10.36660/abc.20220145)
Supplement: Related texts

# Reply: Carta-resposta

**Authors:** Marcio Sommer Bittencourt

Gostaríamos de agradecer pelos comentários a respeito do nosso artigo. Como deixamos claro em nosso artigo, o objetivo foi investigar a associação da razão neutrófilo-linfócito com aterosclerose de aorta abdominal em pacientes assintomáticos.^1^ Apesar de a relação ter sido mais comumente descrita em quadros de instabilidade de placa, publicações prévias reportam seu aumento em associação com doença coronariana estável.

Como apontado, também reconhecemos que a ultrassonografia abdominal tem limitações na avaliação da aterosclerose de aorta. No entanto, devido à rotina de investigação de rastreamento de pacientes assintomáticos, particularmente em grandes bancos de dados como o de nosso recente estudo, este é o único método que pode ser utilizado em larga escala com baixo custo e risco para os pacientes.

Quanto à modelagem proposta na carta, discordamos do ponto de vista dos autores da carta. Modelos de Cox são modelos de sobrevida e somente podem ser utilizados com variáveis com tempo até o desfecho. No caso de coletas simultâneas, não há tempo até o evento e a modelagem recomendada para desfechos binários é a realizada com regressão logística. A referência citada discute sobre estimativa de prevalência, o que não foi realizado em nosso estudo.

A despeito das adequadas críticas propostas, acreditamos que nosso estudo fornece informações inovadoras que contribuem para o conhecimento científico na área.

## References

[1] Marin BS, Ceseria F, Laurinavicius AG, Santos RD, Bittencourt MS (2022). Razão Neutrófilo-Linfócito e Aterosclerose da Aorta Abdominal entre Indivíduos Assintomáticos. Arq Bras Cardiol.

# Reply

**Authors:** Marcio Sommer Bittencourt

We would like to express our gratitude for the comments regarding our article. As stated in our article, the objective was to investigate the association of the neutrophil-to-lymphocyte ratio with abdominal aortic atherosclerosis in asymptomatic patients. ^1^ Although this association has been more frequently described in cases of plaque instability, previous publications have reported its increase in association with stable coronary disease.

As indicated, we also recognize that abdominal ultrasound has limitations in the assessment of aortic atherosclerosis. However, due to the investigation routine for screening asymptomatic patients, especially in large databases such as that of our recent study, this was the only method that could be used on a large scale with low cost and low risk to patients.

With respect to the model proposed in the letter, we disagree with the point of view of the authors of the letter. Cox models are survival models, and they can only be used with time-to-outcome variables. In the case of simultaneous collection, there is no time to event, and the model recommended for binary outcomes is the one with logistic regression. The cited reference discusses the prevalence estimate, which was not performed in our study.

Notwithstanding the relevant points of criticism raised, we believe that our study provides innovative information that contributes to scientific knowledge in the area.
